# The relationship between public service motivation and turnover intention: the mediating role of work stress and task performance

**DOI:** 10.1265/ehpm.22-00045

**Published:** 2022-07-14

**Authors:** Huanhuan Jia, Shang Gao, Panpan Shang, Peng Cao, Jianxing Yu, Xihe Yu

**Affiliations:** School of Public Health, Jilin University, Changchun City, Jilin Province, China

**Keywords:** Turnover intention, Work stress, Task performance, Public service motivation, Medical staff

## Abstract

**Background:**

The shortage of health care workforce is a common problem all over the world and one of the main reasons for the shortage is the high turnover rate. Based on the characteristics of medical work, this study explored the relationship among public service motivation (PSM), work stress, task performance and turnover intention.

**Methods:**

Medical personnel in public hospitals were selected by stratified random sampling in Jilin province of China and validated scales from previous studies were applied to measure the variables. Besides, a structural equation model of turnover intention was constructed to demonstrate the relationship.

**Results:**

A total of 3191 valid questionnaires were collected. The results showed that the score of turnover intention was 2.02 ± 1.13. There are significant differences in turnover intention among medical staff of different genders and departments. At the same time, PSM had direct and negative effects on the turnover intention (*β* = −0.292, *P* < 0.001), work stress had direct and positive effects on the turnover intention (*β* = 0.479, *P* < 0.001), whereas task performance had no significant effect on turnover intention (*β* = 0.044, *P* < 0.142). The results showed an acceptable fit model.

**Conclusion:**

The greater the PSM, the lower the turnover intention, and the higher the work stress, the higher the turnover intention. In addition, work stress and task performance play a mediating role between PSM and turnover intention. This paper provides theoretical support for the measures to reduce the turnover intention of medical staff.

## 1. Background

### 1.1 Background

Reports from the Global Health Workforce Alliance (GHWA) and World Health Organization (WHO) show that it is difficult for health posts to attract and retain a heath care workforce, so health care workforces are lacking worldwide [1]. The shortage of a health care workforce is not only found in developing countries [2, 3], but also in developed ones [4]. According to the CIA World Factbook data, China only had 19.8 physicians per 10,000 people in 2017, below the WHO benchmark [5]. As a developing nation with a large population, China faces multiple problems such as demographic changes, population aging, a heavy burden of non-communicable diseases, and various emerging and emergent public health threats [6]. The abovementioned questions led to a dramatic rise in the demand for health services, and the phenomenon of “difficult to see a doctor” became extremely acute and prominent. One salient reason for “difficult to see a doctor” is the shortage of the health care workforce [7]. In addition, due to the coronavirus prevention and control requirements, there is a need for more health care professionals and services.

China does not lack a “reserve” medical staff, but it cannot effectively transform and retain medical staff. Between 2005 and 2014, China produced 4727,977 clinical medicine graduates but only registered 75,223 clinicians [8]. This partly reflects the fact that retaining doctors has become one of the major challenges facing the Chinese health care system [9]. Because of the long period and high costs of training, the shortage of doctors will become more severe because there is a high turnover rate [10]. The situation of nurses is also not optimistic. According to the China Medical and Health Development Report 2015, 56.94% of Chinese nurses are willing to leave their jobs [11]. The departure of medical staff will lead to serious consequences and will bring property losses to medical and health institutions, impact team morale, increase the workload, create poor quality of care, strain doctor–patient relationships, and escalate medical error claims [10, 12, 13].

According to the theory of behavioral planning, behavioral intention can predict actual behavior well [14], and since turnover intention is usually considered the most effective indicator of employee turnover intention, it makes sense to focus on this element [15–17], and identifying the factors that influence turnover intention can help us reduce health care workers’ turnover intention to effectively control the actual turnover rate of medical staff.

Scholars generally accept that turnover intention is an antecedent variable and the final state of withdrawal cognition before actual turnover. The antecedents of turnover intention can be divided into macrocosm and microcosm factors. Macrocosm factors include the level of employment and unemployment, the level of the social economy, laws, regulations, and the social security system. Microcosmic factors include the factors of organization, the individual, and so on. Research on the influencing factors of turnover intention among health care staff has mostly focused on individual characteristics, attitudes and behaviors; examples include personality traits, life traits, job satisfaction, job performance, burnout, and work stress. Hayes, based on a review of a large number of studies, attributed the factors affecting nurses’ turnover intention to organizational factors, workload, stress, burnout, management, empowerment, and individual factors [18]. During the COVID-19 pandemic, surveys of turnover intention have focused on satisfaction and mental health [19, 20]. Domestic scholars’ research on the turnover intention of Chinese medical and nursing staff has also mainly examined satisfaction, burnout, and stress [21, 22].

However, previous studies have ignored the uniqueness of the health care industry; compared with other fields, the health care sector is a social welfare industry that has the attributes of a public service. Perry and Wise developed the concept of public service motivation (PSM) to understand the behavior of public organizations and the management of staff, such as public hospitals [23]. Therefore, this paper scrutinizes the relationship between PSM and turnover intention based on the occupational attributes of health care staff. PSM is a prosocial behavior oriented by altruism, the inclination to serve others and society, and being willing to sacrifice one’s personal interests to some extent. This connotation dovetails with the virtues and character strengths that a socially recognized health care provider should possess such as compassion, altruism, and benevolence [24]. At the same time, in the prevention and control of COVID-19, the importance of medical staff is generally recognized by society and the public, and there are higher expectations for the performance of medical staff in their tasks. The self-expectations of medical staff are also higher, but people with high expectations do not always show improvement in performance. Instead, excessive expectations can lead to stress-based reactions such as insomnia and even withdrawal behavior [25]. Based on the above discussion, we examined the relationship between PSM, work stress, task performance, and turnover intention.

### 1.2 Theoretical hypotheses

#### PSM and turnover intention

Some scholars believe that employees with high levels of PSM may seek specific professions, especially the medical profession, whose aim is to help others [26]. For the entire public health service system, the efficiency and quality of health care workers as providers of health care services are directly related to public health services, and even to overall public health and the safety of public services [27]. For individual health care workers, PSM is an altruistic desire to serve the common good, to serve others, and to help patients and their families, regardless of financial or other incentives. This desire naturally arouses staff’s emotional motivations of compassion and sacrifice in their interactions with patients [28], which may promote staff retention. Naff and Crum (1999) demonstrated that employees with higher PSM are more satisfied with their jobs and less willing to leave [29].

We therefore proposed the following:

**Hypothesis 1 (H1):** *PSM* has a negative effect on *turnover intention*.

#### Work stress and turnover intention

Most studies define work stress as a psychosomatic emotional response that arises from a mismatch between job demands and one’s abilities, resources and needs [30, 31]. Health care workers in China have long been under tremendous pressure. Motivation orientation can be divided into autonomy orientation and control orientation [32, 33]. Individual motivation orientation not only influences the individual’s stress level, but also the individual’s response to stress [34]. Staff under severe work stress are more likely to be distracted in the performance of their duties as a result of control orientation [35], and the adoption of control orientation in response to stress will lead to a decrease in the effectiveness of the organization’s work and the quality of its medical care [36], higher patient mortality and burnout, absenteeism, low job satisfaction, and turnover intention among health care workers [37]. Individuals with autonomy orientation, on the other hand, tend to view stressors as challenges rather than threats and thus to react less defensively [38]. As an altruistic individual motivation, PSM can be classified as autonomy orientation. Bangcheng Liu et al. (2014) showed that PSM can mitigate the negative effects of stressors on individuals’ physical and mental health; moreover, employees with high PSM tend to have better mental health than those with low PSM [39] and are less likely to leave.

Based on the above discussion, we formulated the following hypotheses:

**Hypothesis 2 (H2):** *Work stress* has a positive effect on *turnover intention*.
**Hypothesis 3 (H3):** *PSM* has a negative effect on *work stress*.
**Hypothesis 3a (H3a):** *Work stress* mediates the relationship between *PSM* and *turnover intention*.

#### Task perforce and turnover intention

Many studies on motivation indicate that while some people are selfish and driven by material things, others are moved by the identities and experiences of others [40]. Nurses, for example, tend to exhibit higher levels of empathy through frequent and long-term contact with patients [27]. Through frequent contact with patients during individualized care, nurses tend to show more compassion and more willingness to take care of the sick. This altruistic behavior coincides with the public service motive of “general, altruistic motivation to serve the interests of a community of people, a state, a nation or humankind,” which may drive health care workers to devote more personal time and energy to their work, as well as to strive for better task performance. Task performance is usually defined as a performance indicator that is directly linked to the output of the work and can be evaluated directly based on the results of the work. Task performance is closely tied to the job content of a specific position, but is also closely related to the individual’s ability to complete a task proficiently and his/her work knowledge [41]. According to the theory of person-environment (PE) fit, the consistency between personal and environmental attributes is positively correlated with higher job satisfaction, higher role participation, and lower turnover intention [42]. The person-job (PJ) fit theory also suggests that people’s job satisfaction and performance improve when they choose a job that matches their characteristics and skills. That is, the better a person does at his/her job, the better suited he/she is for the job he/she is currently doing. In addition, people do not usually give up on a job that suits them.

Hence, we made the following assumptions:

**Hypothesis 4 (H4):** *Task performance* has a negative effect on turnover intention.
**Hypothesis 5 (H5):** *PSM* has a positive effect on *task performance*.
**Hypothesis 5a (H5a):** *Task performance* mediates the relationship between *PSM* and *turnover intention*.
**Hypothesis 6 (H6):** *Work stress* has a negative effect on *task performance*.
**Hypothesis 6a (H6a):** *Task performance* mediates the relationship between *work stress* and *turnover intention*.

## 2. Methods

### 2.1 Study design and participants

We collected the data using a questionnaire survey among medical staff in public hospitals in Jilin Province, China. First, we divided all public hospitals in Jilin Province into urban and county-level public hospitals. Because of the high clustering of urban public hospitals, we applied stratified random sampling to select the sample of urban public hospitals on a 1/4 scale stratified by region, type and level. At the county level, we chose the People’s Hospital and the Traditional Chinese Medicine Hospital as the sample of hospitals from each county. After this process, we finally included a total of 109 hospitals, including 80 county-level public hospitals and 29 urban public hospitals. Next, we sampled 30 medical personnel, including doctors and nurses, from each hospital using quota sampling. Finally, trained investigators conducted an on-site questionnaire survey of medical personnel and recovered the questionnaire on the spot. Our study was approved by the Medical Ethics Committee of the authors’ institute (No. 2019-12-03). In addition, during the survey, the investigator fully explained to the medical personnel the purpose of the survey and informed them that they could withdraw at any time.

A total of 3260 questionnaires were distributed, and after removing the unqualified questionnaires, 3191 valid ones were retrieved, with an effective recovery rate of 97.88%.

### 2.2 Measures

We adapted validated scales from previous studies to measure the variables.

#### Work stress (WS)

We obtained items for work stress from the Challenge-and Hindrance-Related Self-Reported Stress Measures (CHSS) developed by Cavanaugh et al [43]. The CHSS includes challenging and hindrance stress. The challenging stressors were defined as work-related demands or circumstances with potential benefits for the individual, and consisted of six items. Hindrance stressors were defined as work-related demands or circumstances that limit or interfere with an individual’s work achievements, and consisted of five items. We measured the items using a 5-point Likert scale (1 = no stress; 5 = a great deal of stress). The reliability and validity of the scale have been verified [43]. In addition, Chinese scholars have translated it into Chinese, and it has been shown to be applicable to professional groups through investigations [44]. In this study, the Cronbach’s alpha of WS was 0.933, and the two subscales were 0.936 and 0.849, respectively.

#### Task performance (TP)

We based items for task performance (TP) on the performance measurement scale [45]. TP contains 5 items that reflect employees’ performance in terms of fulfilling responsibilities, meeting performance requirements, and not neglecting their duties. We gauged the items using a 5-point Likert scale, and higher scores were associated with better job performance. The Cronbach’s alpha of TP in this study was 0.916.

#### PSM

We obtained the measurement items from the PSM scale developed by Perry, which is widely used by scholars [46, 47]. In addition, previous studies have applied it to the Chinese professional population through the translation and revision process. [48] We selected three dimensions of commitment to the public interest (5 items), compassion (4 items) and self-sacrifice (5 items) to form the measurement tool. We measured each item on a 5-point Likert scale ranging from 1 (completely disagree) to 5 (completely agree), with higher scores indicating greater PSM. In this study, the Cronbach’s alpha of PSM was 0.920, and the three subscales were 0.899, 0.838 and 0.796.

#### Turnover intention (TI)

Most of the turnover intention measurements refer to the scale in Mobley’s employee withdrawal behavior model [49]. Based on this scale, Chinese scholars have revised and applied it to study the turnover intention of doctors; the scale has been shown to have reliability and validity [50]. There are three items in TI: “*I had the idea to leave the hospital*,” “*Within a year, I will look for a new job*,” and “*If I had the chance, I would definitely accept a better new job*.” We measured each item using a 5-point Likert scale, and the higher the score, the stronger the turnover intention of the medical personnel. In this study, the coefficient was 0.849.

In addition, for the questionnaire we collected the demographic characteristics of the respondents, including gender, age, marital status, education level, professional title, department, and working years.

### 2.3 Data analysis

We combined descriptive statistics, t-tests, one-way analysis of variance (ANOVA), structural equation modeling (SEM), and mediating effects in the analytical strategy. We used descriptive statistics to analyze the demographic characteristics of the medical personnel, including gender, age, marital status, education level, professional title, department, and working years. We applied t-tests and ANOVA to describe the differences in the turnover intention of respondents with different characteristics. We used Pearson’s correlation analysis to test the correlations among the variables. We employed Amos 24.0 to construct a model to test the hypotheses. We used the maximum likelihood method in the parameter estimation. We adopted the following criteria to evaluate the model’s fitness [51, 52]:

(1) the chi-square test;
(2) root mean square error of approximation (RMSEA ≤ 0.08);
(3) the comparative fit index (CFI ≥ 0.90);
(4) the Tucker Lewis Index (TLI ≥ 0.90);
(5) the incremental fit index (IFI ≥ 0.90);
(6) the normed fit index (NFI ≥ 0.90); and
(7) the standardized root mean-square residual (SRMR ≤ 0.05).

Finally, we analyzed the mediating effects of work stress and task performance using the bootstrapping technique. All statistical tests were two-sided, with the level of significance set at 0.05.

## 3. Results

### 3.1 Demographic characteristics

Of the 3191 participants, 69.73% (n = 2225) were women, and the mean age was 37.54 years (SD = 8.86). The majority were married (79.72%, n = 2544). Approximately 69.10% (n = 2205) had a college degree or higher. Most participants had junior or middle professional titles (68.41%, n = 2183), and only 4.51% (n = 144) had senior professional titles. These medical personnel were mainly from the departments of internal medicine or surgery, and had worked in hospitals for an average of 12.5 years (SD = 9.48). The demographic characteristics of the participants are shown in Table 1.

**Table 1:** Demographic characteristics and turnover intention of medical personnel

**Variables**	**N (%)**	**TI**	***P***
Gender			
Male	966 (30.3)	2.24 ± 1.20	<0.001
Female	2225 (69.7)	1.93 ± 1.09	
Age			
≤30	846 (26.5)	2.01 ± 1.12	0.184
31–40	1218 (38.2)	2.06 ± 1.15	
41–50	887 (27.8)	1.96 ± 1.12	
≥51	240 (7.5)	2.09 ± 1.13	
Marital status			
Unmarried	550 (17.2)	2.13 ± 1.17	0.071
Married	2544 (79.7)	2.01 ± 1.13	
Divorced	72 (2.3)	1.88 ± 1.04	
Other	25 (0.8)	1.93 ± 1.22	
Education			
High school and below	178 (5.6)	1.91 ± 1.17	0.071
Junior college	808 (25.3)	1.96 ± 1.13	
College	1917 (60.1)	2.05 ± 1.14	
Master’s degree and above	288 (9.0)	2.11 ± 1.08	
Professional title			
Senior	144 (4.5)	2.15 ± 1.17	0.194
Sub-senior	633 (19.8)	2.04 ± 1.14	
Middle	978 (30.7)	2.06 ± 1.13	
Junior	1205 (37.8)	1.97 ± 1.12	
None	231 (7.2)	2.04 ± 1.16	
Department			
Internal medicine	1083 (33.9)	2.05 ± 1.14	0.007
Surgery	625 (19.6)	2.13 ± 1.18	
Gynecology	181 (5.7)	1.82 ± 1.05	
Pediatrics	135 (4.2)	2.14 ± 1.18	
Traditional Chinese medicine	136 (4.3)	2.03 ± 1.10	
Preventive medicine	18 (0.6)	1.83 ± 0.98	
Other	1013 (31.7)	1.95 ± 1.11	
Working years			
≤5	943 (29.6)	1.99 ± 1.09	0.107
6–15	1205 (37.8)	2.08 ± 1.16	
16–25	624 (19.5)	2.02 ± 1.15	
≥26	419 (13.1)	1.95 ± 1.11	
Total	3191 (100.00)	2.02 ± 1.13	

**Note:** TI, turnover intention.

### 3.2 Results of turnover intention

The turnover intention of the participants was 2.02 ± 1.13. The difference in turnover intention between males and females was statistically significant, and the turnover intention of males (2.24 ± 1.20) was higher than that of females (1.93 ± 1.09). The turnover intention of medical staff in different departments was also significantly different, and medical personnel from pediatric (2.14 ± 1.18) and surgical (2.13 ± 1.18) departments had higher turnover intentions than those from other departments. However, there were no significant differences in turnover intention for age, marital status, education level, professional title, or years of work experience. The turnover intention scores of medical personnel with different characteristics are shown in Table 1.

### 3.3 Results of correlations analysis

The scores of work stress, task performance, and PSM are shown in Table 2. The score of PSM was 4.46 ± 0.71, of which the score of commitment to the public interest was 4.34 ± 0.70, the score of self-sacrifice was 3.90 ± 0.83 and the score of compassion was 4.20 ± 0.71. The score of work stress was 2.10 ± 0.85, including 2.27 ± 0.98 for challenging stress and 1.90 ± 0.85 for hindrance stress. Finally, the task performance score was 4.46 ± 0.64. The results of the correlation analysis show that PSM was negatively correlated with work stress (*r* = −0.362, *p* < 0.01) and turnover intention (*r* = −0.401, *p* < 0.01) and positively correlated with task performance (*r* = 0.565, *p* < 0.01). Work stress was positively correlated with turnover intention (*r* = 0.463, *p* < 0.01) and negatively correlated with task performance (*r* = −0.283, *p* < 0.01). Task performance was negatively correlated with turnover intention (*r* = −0.268, *p* < 0.01).

**Table 2:** Correlations among public service motivation, task performance, work stress, turnover intention.

**Variables**	**Mean**	**SD**	**PSM**	**WS**	**TP**	**TI**
PSM	4.46	0.71	1			
WS	2.10	0.85	−0.362**	1		
TP	4.46	0.64	0.565**	−0.283**	1	
TI	2.02	1.13	−0.401**	0.485**	−0.268**	1

**Note:** PSM, public service motivation; TP, Task performance; WS, Work stress; TI, turnover intention; **P < 0.01.

### 3.4 Analytical results of the hypothesized model

We established a structural equation model to verify the theoretical hypothesis and to explore the interrelationships among the variables. We applied maximum likelihood to fit the data, and we calculated the *t* and *p* values for each path. Table 3 describes the direct and indirect effects of each variable on turnover intention.

**Table 3:** Results of the effects on task performance.

**Path**	**Effect**	**Coefficient**	**Boot SE**	**Z**	**P**	**Bias-Corrected 95%CI**		**Percentile 95%CI**	
	
						**Lower**	**Upper**	**Lower**	**Upper**
PSM → TI	Direct	−0.294	0.032	−9.188	<0.001	−0.356	−0.231	−0.357	−0.233
	Indirect1	0.029	0.019	1.526	0.142	−0.009	0.067	−0.008	0.067
	Indirect2	−0.205	0.013	−15.769	<0.001	−0.231	−0.180	−0.230	−0.180
	Total	−0.470	0.019	−24.737	<0.001	−0.506	−0.431	−0.506	−0.431
TP → TI	Direct	0.047	0.030	1.567	0.142	−0.015	0.105	−0.013	0.106
WS → TI	Direct	0.475	0.021	22.619	<0.001	0.433	0.517	0.433	0.516
	Indirect	−0.003	0.002	−1.500	0.13	−0.009	0.000	−0.008	0.001
	Total	0.473	0.021	22.524	<0.001	0.431	0.514	0.430	0.513

**Note:** PSM, public service motivation; TP, Task performance; WS, Work stress; TI, turnover intention; Indirect1, PSM → TP → TI; Indirect2, PSM → WS → TI.

**Fig. 1:**
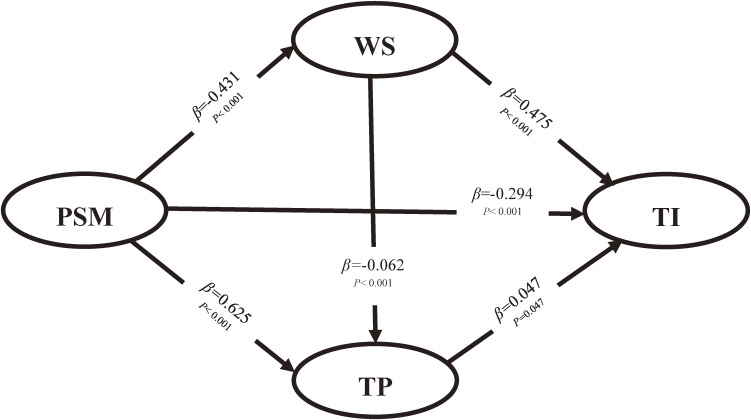
The hypothetical structural equation model. **Note:** TP, Task performance; WS, Work Stress; PSM, Public Service Motivation; TI, Turnover Intention

PSM directly and negatively affected turnover intention (*β* = −0.294, *P* < 0.001), which meant that the higher the PSM, the lower the turnover intention of medical personnel. However, work stress directly and positively affected turnover intention (*β* = 0.475, *P* < 0.001), indicating that the greater the work stress, the higher the turnover intention. Therefore, both H1 and H2 were supported. Although the *P* of the path coefficient for task performance and turnover intention was less than 0.05, whereas it was close to 0.05, and the 95% CI of Bias-Corrected method and Percentile method showed that their confidence intervals contained 0. Therefore, we thought that task performance did not have a significant influence on turnover intention, so H4 was not supported. In addition, the higher the PSM, the lower the work stress, and the better the task performance (*β* = −0.431; *P* < 0.001; *β* = 0.625; *P* < 0.001), so H3 and H5 were supported. The higher the work stress, the worse the task performance (*β* = −0.062; *P* < 0.001), so H6 was also supported. As shown in Fig. 1.

With regard to the mediating effects, we adopted the bootstrapping technique in AMOS to explore the mediating role of work stress and task performance, and we obtained the 95% confidence intervals of the indirect effects with 5000 bootstrap resamples. PSM significantly affected the turnover intention of medical personnel through work stress (*β* = −0.205; *P* < 0.001), whereas the mediating effect of task performance was not significant (*β* = −0.029; *P* = 0.142). In addition, the effect of work stress on turnover intention through task performance was not statistically significant. Therefore, H3a was supported, whereas H5a and H6a were not. Further, the total effects of PSM and work stress on turnover intention were −0.470 and 0.473, respectively. Table 4 shows an acceptable fitting model.

**Table 4:** The fit of the structural equation model

**Model**	**SRMR**	**RMSEA**	**CFI**	**TLI**	**IFI**	**NFI**
Reference	<0.05	<0.08	>0.9	>0.9	>0.9	>0.9
Model	0.0432	0.05	0.975	0.968	0.975	0.972

## 4. Discussion

We examined the factors influencing the turnover intention of health care workers in public hospitals in China. We used standardized scales for data collection and set up a reasonable structural equation model to study the relationships among the PSM, work stress, task performance, and turnover intention of medical staff in public hospitals in China. The model ended up showing good explanatory power.

Turnover intention differed by gender, and male health care workers have higher intention for turnover. We think there are factors that contribute to higher turnover intention for male. First, male health care workers are under greater stress than female. This inference has been confirmed by the conclusions of previous studies [53]. Second, male and female are differing in values. Traditionally, men have stronger achievement motivation and sense of adventure [54]. From this perspective, male medical workers may be more willing to change jobs in order to realize their pursuit of better work, while female medical workers are more likely to work stably in one unit. Third, male health care workers may face worse doctor-patient relationship than female workers. Gender is one of the many factors that impact the doctor–patient interaction [55]. Because of different doctor-patient communication style, diagnostic process style and treatment style, some studies have showed that female physicians face better doctor-patient relationship and higher patient satisfaction for female physicians is more patient-oriented than that of male physicians [56]. In addition, male health care worker also experience work-family imbalances. Generally, we believe that female medical workers have more serious work family imbalance [57], which is certainly an undeniable fact, but this does not mean that families put less pressure on male health care workers than women. For example, studies have found that the working hours of female doctors will be greatly reduced due to child care, but the working hours of male doctors will lead to an increase in working hours [58]. From this, we may infer that under the condition of low overall income of Chinese doctors, it is reasonable for Chinese male health care workers with higher pressure to support their families to have higher willingness to leave. Therefore, we suggest taking gender specific measures to reduce turnover intention. For male health care workers, increasing salary, setting scientific and reasonable promotion methods, training sympathy ability and improving communication ability can effectively reduce the turnover intention of male health care workers. For female health care workers, providing more emotional support, setting up work hours that can balance the family and scientific performance evaluation methods can give them greater help.

Turnover intention is affected by the department of the hospital where the medical staff are located. We believe that this may be due to the different working environment and risk factors faced by the medical staff in different departments. We found that pediatrics is the department with the strongest turnover intention, which is consistent with previous studies [59]. We infer that it may be because pediatric patients are more difficult to communicate, and parents are worried that their children are prone to overreact to pediatric medical staff [60]. Therefore, we believe that the measures to reduce the turnover of pediatric medical staff should focus on emotional counseling, personal safety protection and salary improvement. Surgery is the second most willing department for medical staff to leave, which is consistent with previous studies [61]. Studies believe that the reason why surgical medical workers have the idea of leaving work is working hours. Due to the long working hours and heavy tasks of surgery, many doctors respond that the time left for family and personal hobbies is very limited [62, 63]. Therefore, we suggest that when proposing measures to reduce the surgeon’s willingness to leave, we can tilt the policy to more reasonable working hours, so as to help the surgeon balance work and personal life. Dissatisfaction with the work of general physicians is a common phenomenon [64]. They are dissatisfied with their income and the lack of control over their practices, with more time spent on administrative and business aspects and less time available to see patients [65–68]. Therefore, we believe that in reducing the turnover intention of medical staff, we should focus on improving the salary of medical staff, optimizing procedures, improving time efficiency and improving the participation of medical staff in medical practice. There are few studies on the turnover intention of medical staff in traditional Chinese medicine departments. According to what we learned in the process of investigation and interview, it is inferred that the high turnover intention of traditional Chinese medicine departments may be related to the trend of “emphasizing western medicine and neglecting traditional Chinese medicine” in China for many years, followed by the low salary of traditional Chinese medicine departments compared with others. In view of the difficulties faced by traditional Chinese medicine departments, we believe that we should pay more attention to traditional Chinese medicine departments, strengthen the excavation and standardization of traditional Chinese medicine treatment methods, and improve the salary and attention of traditional Chinese medicine practitioners.

The mean turnover intention score calculated in this paper (mean = 2.02, SD = 1.13) is basically consistent with the results of other studies focusing on the turnover intention of health care workers in China. For example, Ting Cao et al. (2020) reported a turnover intention score of 2.21 (SD = 0.75) in a study of 569 registered nurses in Beijing, China [69]. Yuyin Xiao et al. (2021) reported a turnover intention score of 2.30 (SD = 1.03) in a study of health workers in 16 districts of Shanghai [70].

First, work stress positively affected the turnover intention of health care workers. Higher work stress leads to organizational inefficiency, absenteeism due to illness, increased health care costs, and a high turnover of staff [71]. This article is based on China’s national conditions. Long training periods, long-term deteriorating doctor–patient relationships [72], low incomes, long working hours, high work intensity, high occupational risk, and low social respect are the main reasons for the great pressure on Chinese medical staff. Therefore, we suggest that hospitals’ internal human resource management reduce the work pressure on medical staff from two angles: material and emotional support. Pay reform, for example, would boost the incomes of health care workers. Relevant departments should also issue pertinent policies and laws to prevent and legislate workplace violence and malignant medical injuries to enhance the social respect of medical staff. The buffer theory of social support indicates that others are a primary source of social support because they buffer against the stresses of life and/or help people to better manage stress. Therefore, we also call on the family and friends of medical staff to provide more emotional support to them.

Second, the turnover intention of health care workers is influenced by PSM, and the turnover intention of health care workers with higher PSM is lower [29]. Similar to the results of Bright (2008), employees with higher levels of PSM have better job satisfaction and lower turnover intention when they conform to the characteristics of public organizations. We also found that PSM can influence the turnover intention of health care workers by influencing their work pressure. The transactional theory of stress implies that different employees have different responses to stress. Public officials with higher PSM are less likely to respond to stress in a negative way [39], so the higher the level of PSM, the lower the work stress of health care workers. Based on our results, we recommend paying attention not only to the training of clinical skills, but also to the cultivation of empathy and dedication. Hospitals should pay more attention to PSM when recruiting new staff, and should regard PSM as part of organizational culture. In this way, the cultivation of PSM can be facilitated throughout the entire careers of health care workers to maximize the effect of high-level PSM to reduce turnover intention.

However, the task performance of medical staff has no effect on turnover intention, which may be related to the characteristics of medical work. Medical work has unified rules and regulations, operating norms and clinical practice guidelines, and medical personnel are strictly required to follow the correct steps and norms for practical work. Andersen’s (2009) research supports this inference, and the performance and behavior of health care workers managed by professional norms are similar, even when they have different incentives [26].

We propose exploring the essential meaning of medical services, improving medical education, inspiring a sense of morality and compassion among medical staff, and enhancing their level of PSM to reduce the turnover intention. There are few studies on the turnover intention of medical staff from the perspective of public management, so this paper can fill the gap to some extent. In addition, the shortage of, and increasing pressure on, medical staff are the two most serious problems against the background of coronavirus epidemic prevention and control. Our results are more widely applicable than those of other studies that focus on a single population, can provide a reference for hospital human resource management from a comprehensive standpoint, and have strong application significance. However, we have to admit the limitations of this paper. First, this study was limited to public hospitals in Jilin Province, so the results may not be generalizable. Second, the study was a cross-sectional one and was unable to reflect the causal relationship between variables.

## 5. Conclusion

Given the severe shortage of health care workers worldwide, we explored the relationship between turnover intention and PSM, task performance, and work stress in Chinese public hospitals. We found that the greater the PSM, the lower the turnover intention, whereas the higher the work stress, the higher the turnover intention. In addition, work stress and task performance play intermediary roles between PSM and turnover intention. This paper provides a useful reference for creating measures to reduce the turnover intention of medical staff.

## Abbreviations

GHWA: Global Health Workforce Alliance
WHO: World Health Organization
PSM: Public Service Motivation
PE: Person-Environment
PJ: Person-Job
WS: Work Stress
TP: Task Performance
TI: Turnover Intention
ANOVA: one-way analysis of variance
SEM: Structural Equation Modeling
RMSEA: Root Mean Square Error of Approximation
CFI: Comparative Fit Index
TLI: Tucker Lewis Index
IFI: Incremental Fit Index
NFI: Normed Fit Index
SRMR: Standardized Root Mean-Square Residual

## Declarations

### Availability of data and material

Data available on request from authors.

## References

[1] Report WHO. A Universal Truth: No Health without a workforce. 2013:1–104.

[2] Pulla P. Are India’s quacks the answer to its shortage of doctors? BMJ. 2016;352:i291. doi: 10.1136/bmj.i291. 26797712

[3] Gow J, George G, Mutinta G, Mwamba S, Ingombe L. Health worker shortages in Zambia: an assessment of government responses. J Public Health Policy. 2011;32:476–88. doi: 10.1057/jphp.2011.41. 21850054

[4] Zhang X, Lin D, Pforsich H, Lin VW. Physician workforce in the United States of America: forecasting nationwide shortages. Hum Resour Health. 2020;18:8. doi: 10.1186/s12960-020-0448-3. 32029001 PMC7006215

[5] Rao M, Rao KD, Kumar AKS, Chatterjee M, Sundararaman T. India: Towards Universal Health Coverage 5 Human resources for health in India. Lancet. 2011;377:587–98. 21227499 10.1016/S0140-6736(10)61888-0

[6] Campbell J, Buchan J, Cometto G, David B, Dussault G, Fogstad H, . Human resources for health and universal health coverage: fostering equity and effective coverage. Bull World Health Organ. 2013;91:853–63. 24347710 10.2471/BLT.13.118729 PMC3853950

[7] Li HY, Gu SJ, Gong HZ, Zhang R. International Comparison of Health Human Resource Allocation. Chin Cont Decis Conf. 2020:4252–7.

[8] Lien SS, Kosik RO, Fan AP, Huang L, Zhao XD, Chang XJ, . 10-year trends in the production and attrition of Chinese medical graduates: an analysis of nationwide data. Lancet. 2016;388:11.

[9] Wu D, Wang Y, Lam KF, Hesketh T. Health system reforms, violence against doctors and job satisfaction in the medical profession: a cross-sectional survey in Zhejiang Province, Eastern China. BMJ Open. 2014;4:e006431. doi: 10.1136/bmjopen-2014-006431. PMC4281536 25552614

[10] Lu Y, Hu XM, Huang XL, Zhuang XD, Guo P, Feng LF, . The relationship between job satisfaction, work stress, work-family conflict, and turnover intention among physicians in Guangdong, China: a cross-sectional study. BMJ Open. 2017;7:e014894. doi: 10.1136/bmjopen-2016-014894. PMC5566636 28501813

[11] Fang P. China’s Medical and Health Development Report. 2015.

[12] Waldman JD, Kelly F, Arora S, Smith HL. The shocking cost of turnover in health care. Health Care Manage Rev. 2004;29:2–7. doi: 10.1097/00004010-200401000-00002. 14992479

[13] Wan Q, Li Z, Zhou W, Shang S. Effects of work environment and job characteristics on the turnover intention of experienced nurses: The mediating role of work engagement. J Adv Nurs. 2018;74:1332–41. doi: 10.1111/jan.13528. 29350781

[14] Wen T, Zhang Y, Wang X, Tang G. Factors influencing turnover intention among primary care doctors: a cross-sectional study in Chongqing, China. Hum Resour Health. 2018;16:10. doi: 10.1186/s12960-018-0274-z. 29433519 PMC5809822

[15] Tett RP. Meyer JP. Job satisfaction, organizational commitment, turnover intention, and turnover: path analyses based on meta-analytic findings. 1993;46:259–93. doi: 10.1111/j.1744-6570.1993.tb00874.x.

[16] Carsten JM, Spector PE. Unemployment, job satisfaction, and employee turnover: A meta-analytic test of the Muchinsky model. J Appl Psychol. 1987;72:374–81. doi: 10.1037/0021-9010.72.3.374.

[17] Steel RP, Ovalle NK. A review and meta-analysis of research on the relationship between behavioral intentions and employee turnover. J Appl Psychol. 1984;69:673–86.

[18] Hayes LJ, O’Brien-Pallas L, Duffield C, Shamian J, Buchan J, Hughes F, . Nurse turnover: a literature review - an update. Int J Nurs Stud. 2012;49:887–905. doi: 10.1016/j.ijnurstu.2011.10.001. 22019402

[19] Zhang SX, Chen J, Afshar Jahanshahi A, Alvarez-Risco A, Dai H, Li J, . Succumbing to the COVID-19 Pandemic-Healthcare Workers Not Satisfied and Intend to Leave Their Jobs. Int J Ment Health Addict. 2021. doi: 10.1007/s11469-020-00418-6. PMC7790354 33437225

[20] Labrague LJ, de Los Santos JAA. Fear of COVID-19, psychological distress, work satisfaction and turnover intention among frontline nurses. J Nurs Manag. 2021;29:395–403. doi: 10.1111/jonm.13168. 32985046 PMC7537256

[21] Wen T, Zhang Y, Wang X, Tang G. Factors influencing turnover intention among primary care doctors: a cross-sectional study in Chongqing, China. Hum Resour Health. 2018;16:10. doi: 10.1186/s12960-018-0274-z. 29433519 PMC5809822

[22] Liu W, Zhao S, Shi L, Zhang Z, Liu X, Li L, . Workplace violence, job satisfaction, burnout, perceived organisational support and their effects on turnover intention among Chinese nurses in tertiary hospitals: a cross-sectional study. BMJ Open. 2018;8:e019525. doi: 10.1136/bmjopen-2017-019525. PMC6009508 29886440

[23] Perry JL, Wise LR. The Motivational Bases of Public-Service. Public Adm Rev. 1990;50:367–73. doi: 10.2307/976618.

[24] Huber A, Strecker C, Kachel T, Hoge T, Hofer S. Character Strengths Profiles in Medical Professionals and Their Impact on Well-Being. Front Psychol. 2020;11:566728. doi: 10.3389/fpsyg.2020.566728. 33424679 PMC7786021

[25] Li Y, Li N, Wu M, Zhang M. The Sustainability of Motivation Driven by High Performance Expectations: A Self-Defeating Effect. 2019;11:4397.

[26] Andersen LB. What determines the behaviour and performance of health professionals? Public service motivation, professional norms and/or economic incentives. 2009;75:79–97. doi: 10.1177/0020852308099507.

[27] Deng JW, Guo YL, Ma TY, Yang TA, Tian X. How job stress influences job performance among Chinese healthcare workers: a cross-sectional study. Environ Health Prev. 2019;24. 10.1186/s12199-018-0758-4 PMC6320635 30611191

[28] Belrhiti Z, Van Damme W, Belalia A, Marchal B. Does public service motivation matter in Moroccan public hospitals? A multiple embedded case study. Int J Equity Health. 2019;18:160. doi: 10.1186/s12939-019-1053-8. 31640674 PMC6805632

[29] Bright L. Does Public Service Motivation Really Make a Difference on the Job Satisfaction and Turnover Intentions of Public Employees. Am Rev Public Adm. 2008;38:149–66. doi: 10.1177/0275074008317248.

[30] Lindholm M. Working conditions, psychosocial resources and work stress in nurses and physicians in chief managers’ positions. J Nurs Manag. 2006;14:300–9. doi: 10.1111/j.1365-2934.2006.00636.x. 16629844

[31] Nakakis K, Ouzouni C. Factors influencing stress and job satisfaction of nurses working in psychiatric units: A research review. Health Sci J. 2008;2.

[32] Ryan RM. Psychological Needs and the Facilitation of Integrative Processes. 1995;63:397–427. doi: 10.1111/j.1467-6494.1995.tb00501.x. 7562360

[33] Ryan RM, Deci EL. Self-determination theory and the facilitation of intrinsic motivation, social development, and well-being. Am Psychol. 2000;55:68–78. doi: 10.1037/0003-066x.55.1.68. 11392867

[34] Weinstein N, Ryan RM. A self-determination theory approach to understanding stress incursion and responses. 2011;27:4–17. doi: 10.1002/smi.1368.

[35] Cohen S. Aftereffects of Stress on Human-Performance and Social-Behavior - a Review of Research and Theory. Psychol Bull. 1980;88:82–108. doi: 10.1037/0033-2909.88.1.82. 7403392

[36] Deng JW, Sun YY, Lei R, Yang TAN. Job Stress and Healthcare Quality among Chinese Healthcare Workers: The Mediating Effects of Public Service Motivation. Am J Health Behav. 2019;43:705–16. doi: 10.5993/Ajhb.43.4.5. 31239014

[37] Janssen E, Van Strydonck I, Decuypere A, Decramer A, Audenaert M. How to foster nurses’ well-being and performance in the face of work pressure? The role of mindfulness as personal resource. J Adv Nurs. 2020;76:3495–505. doi: 10.1111/jan.14563. 32989794

[38] Knee CR, Zuckerman M. A Nondefensive Personality: Autonomy and Control as Moderators of Defensive Coping and Self-Handicapping. J Res Pers. 1998;32:115–30. doi: 10.1006/jrpe.1997.2207.

[39] Liu B, Yang K, Yu W. Work-Related Stressors and Health-Related Outcomes in Public Service: Examining the Role of Public Service Motivation. Am Rev Public Adm. 2014;45:653–73. doi: 10.1177/0275074014524298.

[40] Paarlberg LE, Lavigna B. Transformational Leadership and Public Service Motivation: Driving Individual and Organizational Performance. Public Adm Rev. 2010;70:710–8. doi: 10.1111/j.1540-6210.2010.02199.x.

[41] Williams LJ, Anderson SE. Job Satisfaction and Organizational Commitment as Predictors of Organizational Citizenship and In-Role Behaviors. J Manage. 1991;17:601–17. doi: 10.1177/014920639101700305.

[42] Boon C, Biron M. Temporal issues in person-organization fit, person-job fit and turnover: The role of leader-member exchange. Hum Relat. 2016;69:2177–200. doi: 10.1177/0018726716636945. 27904171 PMC5117123

[43] Cavanaugh MA, Boswell WR, Roehling MV, Boudreau JW. An empirical examination of self-reported work stress among U.S. managers. J Appl Psychol. 2000;85:65–74. doi: 10.1037/0021-9010.85.1.65. 10740957

[44] Yang T, Ma M, Zhu M, Liu Y, Chen Q, Zhang S, . Challenge or hindrance: Does job stress affect presenteeism among Chinese healthcare workers? J Occup Health. 2018;60:163–71. doi: 10.1539/joh.17-0195-OA. 29269606 PMC5886884

[45] Williams LJAS. Job Satisfaction and Organizational Commitment as Predictors of Organizational Citizenship and In-Role Behaviors. J Manage. 1991.

[46] Perry J. Measuring Public Service Motivation: An Assessment of Construct Reliability and Validity. J Public Adm Res Theory. 1996;6. doi: 10.1093/oxfordjournals.jpart.a024303.

[47] Wright B, Christensen R, Pandey S. Measuring Public Service Motivation: Exploring the Equivalence of Existing Global Measures. Int Public Manage J. 2013;16:197–223. doi: 10.1080/10967494.2013.817242.

[48] Zhu X. Research on the effect of public service motivation on social workers’ work attitude—A Case Study on Youth Affairs Social Workers in Shanghai [Master] 2014.

[49] Mobley WH, Horner SO, Hollingsworth AT. An evaluation of precursors of hospital employee turnover. J Appl Psychol. 1978;63:408–14. 701211

[50] Zhang Y. The model study on the relationship between job satisfaction, career burnout and turnover intention among physicians from urban state-owned medical institutions [Phd]: Fudan University; 2011. 10.1186/1472-6963-11-235 PMC3197494 21943042

[51] Golini N, Egidi V. The Latent Dimensions of Poor Self-Rated Health: How Chronic Diseases, Functional and Emotional Dimensions Interact Influencing Self-Rated Health in Italian Elderly. Soc Indic Res. 2015;128. doi: 10.1007/s11205-015-1033-3.

[52] Fan N. Strategy Use in Second Language Vocabulary Learning and Its Relationships With the Breadth and Depth of Vocabulary Knowledge: A Structural Equation Modeling Study. Front Psychol. 2020;11:752. doi: 10.3389/fpsyg.2020.00752. 32477204 PMC7237738

[53] Wu H, Zhao Y, Wang JN, Wang L. Factors associated with occupational stress among Chinese doctors: a cross-sectional survey. Int Arch Occup Environ Health. 2010;83:155–64. doi: 10.1007/s00420-009-0456-z. 19701645

[54] Zhang Y, Feng X. The relationship between job satisfaction, burnout, and turnover intention among physicians from urban state-owned medical institutions in Hubei, China: a cross-sectional study. BMC Health Serv Res. 2011;11:235. doi: 10.1186/1472-6963-11-235. 21943042 PMC3197494

[55] Bertakis KD. The influence of gender on the doctor-patient interaction. Patient Educ Couns. 2009;76:356–60. doi: 10.1016/j.pec.2009.07.022. 19647968

[56] Lagro-Janssen AL. [Medicine is not gender-neutral: influence of physician sex on medical care]. Ned Tijdschr Geneeskd. 2008;152:1141–5. 18549138

[57] Estryn-Behar M, Fry C, Guetarni K, Aune I, Machet G, Doppia MA, . Work week duration, work-family balance and difficulties encountered by female and male physicians: results from the French SESMAT study. Work. 2011;40 Suppl 1:S83–100. doi: 10.3233/WOR-2011-1270. 22112665

[58] Song J, Cheng TC. How do gender differences in family responsibilities affect doctors’ labour supply? Evidence from Australian panel data. Soc Sci Med. 2020;265:113475. doi: 10.1016/j.socscimed.2020.113475. 33257176

[59] Ghawadra SF, Abdullah KL, Choo WY, Phang CK. Psychological distress and its association with job satisfaction among nurses in a teaching hospital. J Clin Nurs. 2019;28:4087–97. doi: 10.1111/jocn.14993. 31294501

[60] Yang H, Lv J, Zhou X, Liu H, Mi B. Validation of work pressure and associated factors influencing hospital nurse turnover: a cross-sectional investigation in Shaanxi Province, China. BMC Health Serv Res. 2017;17:112. doi: 10.1186/s12913-017-2056-z. 28158979 PMC5292011

[61] Mahoney ST, Strassle PD, Schroen AT, Agans RP, Turner PL, Meyer AA, . Survey of the US Surgeon Workforce: Practice Characteristics, Job Satisfaction, and Reasons for Leaving Surgery. J Am Coll Surg. 2020;230:283–93 e1. doi: 10.1016/j.jamcollsurg.2019.12.003. 31931143

[62] Kao LS, Wilson EB, Anderson KD. Perceptions and predictors of surgeon satisfaction: a survey of spouses of academic surgeons. J Am Coll Surg. 2005;200:684–90. doi: 10.1016/j.jamcollsurg.2005.01.009. 15848358

[63] Shanafelt TD, Balch CM, Bechamps GJ, Russell T, Dyrbye L, Satele D, . Burnout and career satisfaction among American surgeons. Ann Surg. 2009;250:463–71. doi: 10.1097/SLA.0b013e3181ac4dfd. 19730177

[64] Wetterneck TB, Linzer M, McMurray JE, Douglas J, Schwartz MD, Bigby J, . Worklife and satisfaction of general internists. Arch Intern Med. 2002;162:649–56. doi: 10.1001/archinte.162.6.649. 11911718

[65] Lewis CE, Prout DM, Chalmers EP, Leake B. How satisfying is the practice of internal medicine? A national survey. Ann Intern Med. 1991;114:1–5. doi: 10.7326/0003-4819-114-1-1. 1983926

[66] Petersdorf RG, Goitein L. The future of internal medicine. Ann Intern Med. 1993;119:1130–7. doi: 10.7326/0003-4819-119-11-199312010-00011. 8239233

[67] Reames HR, Jr., Dunstone DC. Professional satisfaction of physicians. Arch Intern Med. 1989;149:1951–6. 2774775

[68] Wahls TL, Olson KA, Turney SL. Practice satisfaction and dissatisfaction in general internal medicine departments of large multispecialty clinics. J Gen Intern Med. 1993;8:578–9. doi: 10.1007/BF02599644. 8271093

[69] Cao T, Huang XX, Wang LM, Li B, Dong X, Lu H, . Effects of organisational justice, work engagement and nurses’ perception of care quality on turnover intention among newly licensed registered nurses: A structural equation modelling approach. J Clin Nurs. 2020;29:2626–37. 32279372 10.1111/jocn.15285

[70] Xiao Y, Dong M, Shi C, Zeng W, Shao Z, Xie H, . Person-environment fit and medical professionals’ job satisfaction, turnover intention, and professional efficacy: A cross-sectional study in Shanghai. PLoS One. 2021;16:e0250693. doi: 10.1371/journal.pone.0250693. 33905430 PMC8078800

[71] AbuAlRub RF. Job Stress, Job Performance, and Social Support Among Hospital Nurses. J Nurs Scholarsh. 2004;36:73–8. doi: 10.1111/j.1547-5069.2004.04016.x. 15098422

[72] Jing W, Otten H, Sullivan L, Lovell-Simons L, Granek-Catarivas M, Fritzsche K. Improving the doctor-patient relationship in China: the role of balint groups. Int J Psychiatry Med. 2013;46:417–27. doi: 10.2190/PM.46.4.g. 24922991

